# The feasibility and effectiveness of high-intensity boxing training versus moderate-intensity brisk walking in adults with abdominal obesity: a pilot study

**DOI:** 10.1186/2052-1847-7-3

**Published:** 2015-01-16

**Authors:** Birinder S Cheema, Timothy B Davies, Matthew Stewart, Shona Papalia, Evan Atlantis

**Affiliations:** School of Science and Health, University of Western Sydney, Locked Bag 1797, Penrith, 2751 Campbelltown, New South Wales Australia; The National Institute of Complementary Medicine, University of Western Sydney, Campbelltown, NSW 2650 Australia; School of Nursing and Midwifery, University of Western Sydney, Campbelltown, New South Wales Australia; School of Medicine, University of Adelaide, Adelaide, South Australia Australia

**Keywords:** High-intensity interval training, Exercise, Body composition, Weight loss, Fat loss, Exercise, Health, Quality of life

## Abstract

**Background:**

High-intensity interval training (HIIT) performed on exercise cycle or treadmill is considered safe and often more beneficial for fat loss and cardiometabolic health than moderate-intensity continuous training (MICT). The aim of this pilot study was to assess the feasibility and effectiveness of a 12-week boxing training (HIIT) intervention compared with an equivalent dose of brisk walking (MICT) in obese adults.

**Methods:**

Men and women with abdominal obesity and body mass index >25 kg/m^2^ were randomized to either a boxing group or a brisk walking (control) group for 12 weeks. Each group engaged in 4 training sessions per week, equated for total physical activity. Feasibility outcomes included recruitment rates, assessment of training intensities, adherence and adverse events. Effectiveness was assessed pre and post intervention via pertinent obesity-, cardiovascular-, and health-related quality of life (HRQoL) outcomes.

**Results:**

Nineteen individuals expressed an interest and 63% (n = 12) consented. Recruitment was slower than anticipated (1.3 participants/week). The boxing group trained at a significantly higher intensity each week versus the brisk walking group (p < 0.05). Two participants in the boxing group experienced an adverse event; both continued to exercise with modifications to the exercise program. No other adverse events were noted. The boxing group attended more sessions (79% vs. 55%) and had a lower attrition rate (n = 0 vs. n = 2) than the walking group. Analysis of covariance revealed that the boxing group significantly improved body fat percentage (p = 0.047), systolic blood pressure (p = 0.026), augmentation index (AIx; p < 0.001), absolute VO_2max_ (p = 0.015), and Physical Functioning (p = 0.042) and Vitality (p = 0.024) domains of HRQoL over time. The walking group did not improve any clinical outcomes, and experienced a worsening of Vitality (p = 0.043).

**Conclusions:**

Boxing training (HIIT) in adults with abdominal obesity is feasible and may elicit a better therapeutic effect on obesity, cardiovascular, and HRQoL outcomes than an equivalent dose of brisk walking (MICT). Robustly designed randomized controlled trials are required to confirm these findings and inform clinical guidelines and practice for obesity treatment.

**Trial registration:**

Trial registration: ACTRN12615000007538

## Background

Obesity is a major risk factor for many chronic non-communicable diseases, and the prevalence of this condition in the global population has doubled since 1980 [1]. Recent data suggest that approximately 36.9% of men and 29.8% of women worldwide are categorically obese [2]. The high and rising prevalence of obesity is associated with increased rates of cardiovascular diseases, cancers [3], type 2 diabetes and chronic kidney disease [1], placing tremendous strain on healthcare systems and national economies [4]. Individuals with obesity also suffer from low health-related quality of life (HRQoL) [5] and increased mortality [6] versus their healthy peers. Innovative and efficient strategies for treating excess body fat are still needed, and could result in significant health and economic benefits.

Current physical activity guidelines [7, 8] recommend 150 to >250 minutes per week of moderate-intensity continuous training (MICT) such as brisk walking to target overweight/obesity and maintain an optimal body weight. These physical activity guidelines are similar to those recommended by the World Health Organization for general health [9]. However, randomized controlled trials (RCT) suggest that brisk walking interventions (≥12 weeks) elicit only a small beneficial effect on body weight and adiposity outcomes in overweight and obese adults [10–12]. Hence, this modality of exercise, despite being recommended [7, 8], may not be particularly effective for inducing clinically meaningful reductions in body fat.

High-intensity interval training (HIIT) involves alternating brief (6 s-4 min) high intensity (≥75% VO_2max_) and lower intensity workloads or rest throughout an exercise session. This shorter duration of exercise may be a more efficient alternative for eliciting fat loss than MICT. Studies in patients with cardiac and metabolic diseases [13–17] have consistently shown that HIIT (≥12 weeks) performed on a cycle or treadmill ergometer is safe, and can result in greater improvements of cardiorespiratory fitness (VO_2max_), endothelial function (i.e. atherosclerosis), insulin signalling and left ventricular morphology and function versus MICT matched for training load or volume (frequency/duration). Studies have also shown that HIIT can significantly increase HRQoL [13, 18]. There is preliminary evidence suggesting that HIIT can elicit significantly greater reductions of adiposity than MICT in overweight and obese cohorts [19–21]. However, this evidence is less consistent and indicates a need for further research.

Boxing training typically involves high intensity intervals of 2 to 3 minutes combined with shorter intervals of rest. Therefore, boxing training is recognized as a metabolically demanding mode of HIIT [22]. Despite its popularity in practice, no study to date has investigated the use of boxing training as a mode of HIIT for the management of obesity and related health outcomes. Therefore, the aim of this pilot study was to assess the feasibility and effectiveness of a 12-week boxing training (HIIT) intervention compared with an equivalent dose of brisk walking (MICT) in adults with abdominal obesity. Feasibility outcomes included recruitment rates, assessment of training intensities, adherence and adverse events, and effectiveness was assessed pre and post intervention *via* pertinent obesity, cardiovascular, and HRQoL outcomes.

## Methods

### Study design

A parallel group design that randomized participants to a boxing group or brisk walking (control) group was utilized. Outcome measures were assessed prior to and following a 12-week intervention period. Post intervention testing was completed >72 hr after the final exercise session. A blinded assessor with *International Society for the Advancement of Kinanthropometry* (*ISAK*) accreditation collected all anthropometric (obesity) data. Randomization assignments were computer-generated (http://www.randomization.com) and stratified by gender, by an investigator not involved in data collection; assignments were given to participants in sealed envelopes upon the completion of baseline testing. The University of Western Sydney Human Research Ethics Committee approved all procedures, and informed consent was received from all participants.

### Participants

Participants were recruited over a nine-week period from June 3 to August 2, 2013 by means of flyer advertisements, university staff email lists and social media (Facebook). The aim was to recruit two participants per week, on average. *Eligibility criteria*: adult (>18 years); body mass index (BMI) >25 kg/m^2^; abdominal obesity as a risk factor for cardiometabolic disease according to the International Diabetes Federation (i.e. waist circumference >94 cm in men and >80 cm in women) [23]; available to complete four exercise sessions per week; able to communicate in English; willingness and cognitive ability to provide written informed consent. *Exclusion criteria:* physically active (i.e. engaging in greater than 3 sessions of moderate-intensity exercise per week); current or history of ischaemic heart disease, cerebrovascular disease, type 2 diabetes mellitus, advanced metabolic disease (e.g. chronic kidney disease) or uncontrolled pulmonary disease.

### Interventions

#### Boxing

Participants in the boxing group were prescribed four, 50-min sessions of supervised boxing training per week. A boxing instructor assisted in designing the program. All sessions were fully supervised by qualified personnel. The interval-based exercises were preceded by a 5 min warm-up of continuous skipping at a self-selected intensity. Intervals were prescribed at 2:1 (i.e. 2 min of high-intensity activity followed by 1 min of rest (standing or pacing) between intervals and exercises). Three intervals of each of the following five exercises were performed for a total of 30 min of high-intensity effort: (1) heavy bag, (2) focus mitts, (3) circular body bag, (4) footwork drills, and (4) skipping. The total amount of physical activity (excluding warm up and rest periods) was computed as 30 min × 6 metabolic equivalents (MET) per minute = 180 MET min [24]. During the high-intensity bouts, participants were instructed to exercise at a rating of perceived exertion of 15-17/20 (“hard” to “very hard”) with the goal of achieving >75% of age-predicted maximal heart rate (i.e. 220-age; HR_max_).

##### Training intensity

Heart rate was monitored using a Polar Heart Rate monitor (Polar® RS800sd Kempele, Finland) and was recorded at the completion of each high-intensity interval; these values were averaged across each week, and reported as percentage of age-predicted HR_max_.

#### Walking

Participants in the walking group were prescribed four, 50-min sessions of brisk walking per week. These sessions were unsupervised and completed in any location convenient to the participant (e.g. within their own neighbourhood, etc.). Participants were instructed to begin each session with a 5-min gradual warm-up and walk as quickly as possible for the remainder of the session (45 min). The total amount of physical activity (excluding warm up) was computed as 45 min × 4 metabolic equivalents (MET) per minute = 180 MET-min [24].

##### Training intensity

Participants were taught how to manually monitor their heart rate and record the measure pre-, mid- and post-exercise. These data were recorded in a training journal. The mid-exercise readings were averaged across each week, and reported as percentage of age-predicted HR_max_.

### Adherence and adverse events

Adherence was defined as the number of training sessions attempted divided by the number offered multiplied by 100%. Adverse events were documented by means of a structured, open-ended questions administered weekly, in person or *via* telephone or email.

### Clinical outcome measures

#### Obesity outcomes

Waist circumference was measured in a horizontal plane, midway between the inferior margin of the ribs and the superior border of the iliac crest, according to standard protocol [25]. Height and weight were measured with calibrated scale (A&D Company Ltd., Japan) and stadiometer (Holtain Ltd., Crymych, UK), respectively, and BMI was computed from these measures [25]. Six skinfold sites (i.e. triceps, subscapular, supraspinale, abdominal, front thigh and medial calf) were recorded using Holtain callipers (Holtain Ltd., Crymych, UK) if the skinfold was ≤40 mm and Slimguide callipers (Creative Health Products, Plymouth, USA) if the skinfold was >40 mm. Body fat percentage was computed from the six skinfold sites using validated equations [26].

#### Cardiovascular outcomes

Resting blood pressure (systolic/diastolic) and heart rate were assessed manually at the brachial and radial artery, respectively, with the participant in a seated position according to standard protocols [27]. Arterial stiffness was assessed using the SphygmoCor System (AtCor Medical Pty, Sydney, Australia). This system derives the central aortic pressure waveform noninvasively from the pulse pressure recorded at a peripheral site. Participants were seated for a 10-min period, then radial pulse pressure waveform recording were obtained using a hand-held, high fidelity tonometer (Millar Instruments, Houston, Texas). The aortic pressure waveforms are comprised of a forward wave caused by left ventricular contraction and a reflected wave due to backflow arising from regions of increased impedance in the peripheral vessels. The resultant of these two pressures is known as the augmentation index (AIx) which was recorded. The SphygmoCor algorithm normalises AIx to a heart rate of 75 beats per minute. This method is highly reproducible [28] and has been used extensively to evaluate arterial stiffness in healthy [29, 30] and chronically diseased cohorts [31]. Population norms for AIx range from -23.27% to 63.07% in adults (aged 18–86 years) with larger values indicating greater arterial stiffness [32].

Cardiorespiratory fitness (VO_2max_) was assessed *via* indirect calorimetry (Jaeger Metabolic Gas Analysers, Viasys Healthcare, Germany) using a standard ramp protocol on a laboratory treadmill (LE 200 CE, Viasys Health Care, USA). O_2_ and CO_2_ sensors were calibrated prior to each test using high-grade calibration gas with certified gas concentrations (O_2_ = 16%, CO_2_ = 5%, N_2_ = balance). The protocol began at a pre-determined, comfortable walking speed for 3 minutes; the grade was increased by 2% each minute thereafter until volitional fatigue.

#### HRQoL outcomes

The *Medical Outcomes Trust Short Form-36 Health Survey (Version 1.0)* (SF-36) [33] was used to evaluate Physical Functioning, General Health and Vitality domains of HRQoL. Higher scores, ranging from 0–100, denote higher HRQoL.

### Statistical analyses

Analyses were performed using the Statistical Package for the Social Sciences (IBM©, SPSS Version 19.0). All data were inspected visually and statistically for normality (skewness and kurtosis between -1 and +1). Analyses were completed according to intention-to-treat strategy, using the last-observation-carry-forward imputation method for any missing data. Within group changes over time were evaluated by paired t-tests. Group x time effects were determined by analysis of covariance (ANCOVA) of the post-treatment score controlling for the baseline score and potential confounding variables identified by comparing groups at baseline. Cohen’s *d* effect sizes were calculated to explore the effectiveness of the intervention and provide data to inform sample size calculations in future studies. Effect sizes were interpreted according to the conventions of small (0.20), moderate (0.50), or large (0.80). A p value of <0.05 was considered indicative of statistical significance.

## Results

### Recruitment and flow of participants

**Figure 1:**
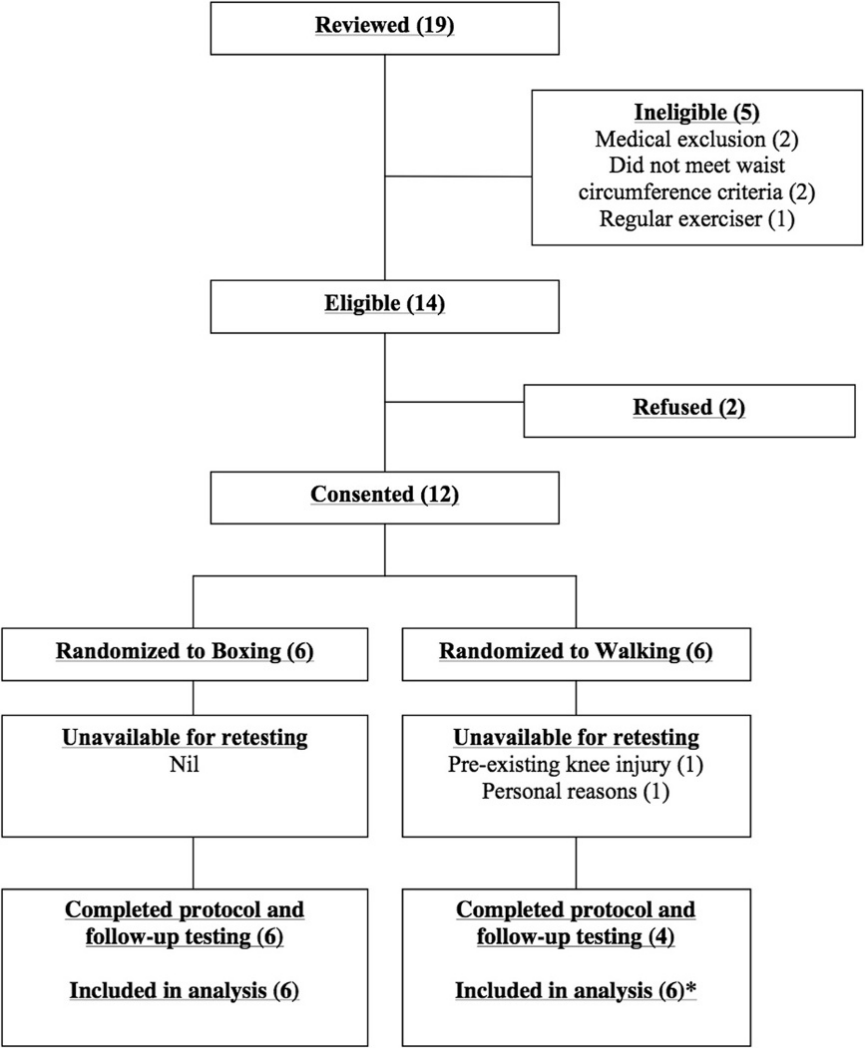
**Flow of participants through the trial.** *Baseline data carried forward for 2 participants lost to follow-up.

Nineteen individuals (n = 19) expressed an interest in the study and five individuals were deemed ineligible due to a medical condition, or not meeting the waist circumference or physical activity criteria (Figure 1). Fourteen individuals were deemed eligible, however two elected not to participate due to inconvenience. Twelve individuals provided written informed consent, completed baseline testing and were randomized into the boxing training (n = 6) or walking (n = 6) groups. Recruitment rate was slower than expected (12 participants/9 weeks of recruitment = 1.3 participants recruited per week). Two female participants in the walking group withdrew: one due to a pre-existing knee injury requiring surgery (week 2) and one for personal reasons (week 5). Baseline data were carried forward for both participants for analyses (intention-to-treat).

### Baseline characteristics

No statistically significant differences were noted between groups at baseline (Table 1). However, due to clinically important differences in age and waist circumference, these variables were included as covariates in all ANCOVA models. The cohort ranged in age from 19 to 72 years, BMI ranged from 26.4 to 40.3 kg/m^2^ and all participants fulfilled the criterion for abdominal obesity [23]. Common comorbidities included hypertension (n = 3) and asthma (n = 2).

**Table 1:** **Baseline characteristics of the total cohort and groups**

Characteristic	Total cohort (n = 12)	Boxing (n = 6)	Walking (n = 6)
Age (y)	39 (17)	43 (19)	36 (15)
Women:Men	7:5	3:3	4:2
Body weight (kg)	90.7 (16.3)	95.7 (21.0)	85.6 (9.1)
Height (cm)	169.6 (8.2)	172.3 (7.7)	166.8 (8.4)
BMI (kg/m^2^)	31.4 (4.4)	32.0 (5.9)	30.8 (2.6)
Waist circumference (cm)	104.7 (14.4)	111.0 (18.0)	98.4 (6.2)
Body fat percentage (%)	35.4 (11.3)	33.5 (10.1)	37.3 (13.1)

Data continuous variables reported as mean (standard deviations).

### Training intensity

**Figure 2:**
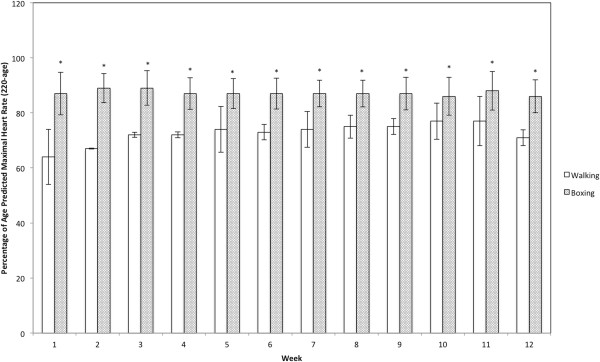
**Mean training heart rate in the boxing and walking groups.** *Significant difference versus the walking group.

Mean training heart rate during the 12-week intervention period ranged from 86-89% and 64-77% of age-predicted HR_max_ in the boxing and walking groups, respectively (Figure 2). The boxing group trained at a significantly higher intensity versus the walking group in each week (p < 0.05).

### Adherence and adverse events

Adherence to training was 79 ± 15% and 55 ± 43% in the boxing and walking groups, respectively, inclusive of the two participants in the walking group who withdrew (Figure 1). Excluding these participants, attendance in the walking group was 82 ± 12%. The difference in attendance between groups was not significant inclusive- (p = 0.24) or exclusive (p = 0.75) of the two participants who dropped out. Five participants in the boxing group and 3 participants in walking group attended more than 70% of their prescribed exercise sessions.

A female participant in the boxing group experienced tennis elbow in week 1 that may have been due to the intervention. She remained in the study by substituting kicking and elbow striking in place of punching. A male participant in the boxing group experienced a strain of the gastrocnemius muscle in week 8, which may have been induced by skipping. The participant substituted rowing for skipping while his calf muscle recovered. Neither participant missed a training session due to their injury. No additional adverse events were noted.

### Outcomes

#### Obesity outcomes

Within and between group analyses are presented in Table 2. The boxing group reduced body fat percentage over time with a small to medium effect that reached statistical significance (Cohen’s *d* = 0.41; p = 0.047). Waist circumference, body mass and BMI was also reduced in the boxing group with small to medium effect (Cohen’s *d* = 0.29-0.48); however, these effects did not achieve statistical significance. The walking group reduced body fat percentage over time with a small effect (Cohen’s *d* = 0.21; p = 0.17) with no other effects noted. No group x time interaction effects were noted for waist circumference, body mass, BMI, or body fat percentage.

**Table 2:** **Summary of within and between group changes on clinical outcomes**

Outcome measures	Boxing (n = 6)					Walking (n = 6)					***P (between groups)***	***Effect size (between groups)***
	Week 0	Week 16	%Change	***P***	Effect size ***(within group)***	Week 0	Week 16	%Change	***P***	Effect size ***(within group)***		
***Obesity outcomes:***												
Waist circumference (cm)	111.1 (18.0)	104.4 (12.2)	-5.3 (7.7)	0.19	0.48	98.4 (6.2)	97.8 (6.7)	-0.6 (2.7)	0.61	0.10	0.86	0.004
Body mass (kg)	95.7 (21.0)	90.8 (15.1)	-4.1 (7.0)	0.23	0.29	85.6 (9.1)	85.4 (9.4)	-0.3 (1.2)	0.63	0.02	0.93	0.001
BMI (kg/m^2^)	32.0 (5.9)	30.5 (4.0)	-4.0 (7.3)	0.25	0.33	30.8 (2.6)	30.7 (3.0)	-0.3 (1.8)	0.78	0.03	1	0
Body fat percentage (%)	33.5 (10.1)	29.5 (11.1)	-13.2 (10.6)	0.047	0.41	37.3 (13.1)	35.0 (11.3)	-5.4 (7.5)	0.17	0.21	0.95	0.004
***Cardiovascular outcomes:***												
Resting HR (beats/min)	66 (4)	62 (6)	-6 (8)	0.10	0.86	75 (12)	74 (11)	-1 (4)	0.43	0.09	0.34	0.13
Resting SBP (mmHg)	137 (12)	123 (8)	-10 (7)	0.026	1.50	127 (4)	129 (8)	+2 (5)	0.45	0.34	0.10	0.33
Resting DBP (mmHg)	89 (8)	82 (9)	-8 (8)	0.074	0.90	89 (7)	89 (5)	0 (7)	1	0	0.16	0.26
Augmentation Index (%)	17.0 (15.4)	7.2 (15.7)	-126.7 (128.4)	<0.001	0.69	12.0 (18.5)	16.8 (12.6)	+41.4 (95.2)	0.41	0.33	0.064	0.41
Pulse Pressure (mmHg)	47 (9)	41 (5)	-10 (23)	0.28	0.90	38 (3)	41 (4)	+9 (14.0)	0.22	0.93	0.36	0.12
VO_2_max (ml/kg min)	27.9 (2.4)	32.5 (5.0)	+16.9 (18.6)	0.06	1.28	29.0 (6.4)	28.8 (8.0)	-1.1 (16.3)	0.93	0.03	0.34	0.13
VO_2_max (L/min)	2.691 (0.688)	2.970 (0.802)	+10.2 (6.7)	0.015	0.41	2.490 (0.691)	2.454 (0.761)	-1.6 (16.5)	0.78	0.05	0.22	0.21
***HRQoL outcomes:***												
Physical Functioning	92.5 (6.1)	96.7 (2.6)	+4.7 (4.4)	0.042	0.98	91.7 (11.7)	90.8 (8.0)	-0.12 (9.3)	0.81	0.10	0.11	0.32
General Health	53.3 (8.8)	65.0 (8.9)	+25.2 (32.1)	0.07	1.45	65.8 (20.1)	62.5 (25.1)	-7.3 (16.8)	0.44	0.15	0.21	0.22
Vitality	50.0 (28.5)	66.3 (191)	+54.8 (49.9)	0.024	0.74	68.8 (32.6)	59.7 (33.8)	-19.1 (28.3)	0.043	0.30	0.02	0.54

Data expressed as mean (standard deviation). *Abbreviations*: *BMI* body mass index, *HR* heart rate, *SBP* systolic blood pressure, *VO*_*2peak*_ peak oxygen consumption. Effect size = Cohen’s *d.*

#### Cardiovascular outcomes

The boxing group reduced resting systolic- and diastolic blood pressure, heart rate, augmentation index, and pulse pressure over time with a medium to large effect (Cohen’s *d* = 0.69-1.50); reductions in AIx (p < 0.001) and systolic blood pressure (p = 0.026) reached statistical significance. By contrast, the walking group unexpectedly increased pulse pressure, AIx, and systolic blood pressure over time with a small to large effect (Cohen’s *d* = 0.33-0.93) though none of these changes reached statistical significance (Table 2). Small to medium group x time interaction effects indicating positive adaptation in the boxing group versus the walking group were noted for AIx (Cohen’s *d* = 0.41; p = 0.064) and resting systolic- (Cohen’s *d* = 0.33; p = 0.10) and diastolic blood pressure (Cohen’s *d* = 0.26; p = 0.16).

The boxing group improved relative- (Cohen’s *d* = 1.28; p = 0.06) and absolute VO_2max_ (Cohen’s *d* = 0.41; p = 0.015) over time with a large and medium effect, respectively. The effect for absolute VO_2max_ was statistically significant. The control group did not improve these measures over time. No group x time interaction effects were noted for VO_2max_ measures.

#### HRQoL outcomes

The boxing group improved Physical Functioning (Cohen’s *d* = 0.98; p = 0.042), General Health (Cohen’s *d* = 1.45; p = 0.07) and Vitality (Cohen’s *d* = 0.74; p = 0.024) domains of HRQoL over time with large effects. The effects for Physical Functioning and Vitality were significant. By contrast, the walking group significantly reduced Vitality over time with a moderate effect (Cohen’s *d* = 0.30; p = 0.043) and did not experience a change in the other two domains. Small to moderate group x time interaction effects favouring the boxing group were noted each of these HRQoL domains (Cohen’s *d* = 0.22-0.54) with the effect for Vitality reaching statistical significance (p = 0.02).

### Sensitivity and post hoc analyses

Group x time sensitivity analyses were performed without imputation of missing data from the two participants in the walking group who withdrew and did not undergo final assessment. The outcomes of these analyses did not differ with primary analyses except that AIx was significantly improved in the boxing group versus the walking group over time (Cohen’s *d* = 0.58; p = 0.046).

To determine the effect of higher adherence on treatment effects, group x time *post hoc* analyses were performed using data from participants who completed >70% of the boxing (n = 5) or walking (n = 3) intervention. The outcomes of these analyses did not differ with the primary analyses.

## Discussion

This is the first pilot study to assess the feasibility and effectiveness of a 12-week boxing training (HIIT) intervention compared with an equivalent dose of brisk walking (MICT) in 12 obese adults. Our recruitment rate was slower than anticipated. There is some evidence to suggest that boxing might be perceived as a high-risk activity [34], and methods to facilitate recruitment and retention, perhaps involving appropriate survey instruments [35] should be considered in the development of future trials. No serious adverse events were noted and the boxing group attended more exercise sessions and had a lower attrition rate than the walking group, suggesting that this form of HIIT may be feasible to administer in this cohort. Limitations of this pilot study included the small sample size (n = 12), lack of a non-exercising control group, the lack of direct supervision of the walking group, and the lack of monitoring of dietary and physical activity changes, which are key confounding variables. These limitations must be considered in the development of future RCT.

### Obesity outcomes

The boxing group significantly reduced body fat percentage (-13.2%; p = 0.047) and experienced a small-to-moderate (non-significant) reduction of waist circumference (-5.3%) body mass (-4.1%) and BMI (-4.0%) over time. Collectively, these adaptations indicate reduced body adiposity, which is associated with a reduced risk of chronic diseases (i.e. insulin resistance, type 2 diabetes, cancers, and cardiovascular diseases) and associated mortality [36–38]. It is difficult to quantify the clinical significance of these changes given our small sample size and pooling of data for men and women. Stevens et al. [39] suggest that ≥5% weight loss is clinically meaningful. The boxing group approached this level of change (-4.1%) while no change was noted in the walking group (-0.3%). A longer training duration (>12 weeks) or more frequent training (>4 sessions/wk) may have induced more favourable adaptation of obesity outcomes.

Studies in overweight and obese participants have consistently shown that HIIT performed on treadmill or cycle ergometer (12–26 weeks) can reduce BMI, waist circumference, body weight and body fat percentage versus a no-exercise control [17]. However, the effect of HIIT versus MICT on adiposity outcomes remains equivocal with many studies demonstrating an equivalent effect [17, 40] and other studies showing a superior [19, 20] or inferior effect [41] of HIIT. These inconsistent data may be due to the heterogeneity of HIIT interventions (e.g. intervals have ranged from 6 s to 4 min [21]), cohorts (e.g. age, level of obesity), and methods use to equate the HIIT and MICT prescriptions across studies. Further research is therefore warranted [21]. No significant group x time interaction effects were noted for obesity outcomes in the present study. However, within group changes suggest a need for a well-powered study of boxing training (HIIT) versus brisk walking (MICT) to explore these comparisons further. Such studies should also investigate the effect of various prescriptions (dosages) of HIIT on these outcomes. Based on the percentage change score data for abdominal obesity (waist circumference) in the boxing group (-5.3 ± 7.7%) and walking group (-0.6 ± 2.7%), we estimate that approximately 50 participants (25 per group) would provide 80% power to detect a statistically significant difference between groups on this outcome measure.

### Cardiovascular outcomes

The boxing group significantly reduced resting systolic blood pressure (-10%; p = 0.026) and AIx (-126.7%; p < 0.001) and increased absolute VO_2max_ (+10.2%; p = 0.015) while experiencing large effects (Cohen’s *d* >0.86) and trends toward reduced resting heart rate (-6%; p = 0.10) and diastolic blood pressure (-8%; p = 0.074) and relative VO_2max_ (+16.9%; p = 0.06). No adaptations were noted in the walking group, and trends toward group x time interaction were noted for AIx (p = 0.064), and resting systolic- (p = 0.10) and diastolic blood pressure (p = 0.16).

Our findings suggest that the boxing intervention induced favourable adaptations of the central and peripheral cardiovascular system. HIIT has consistently been shown to increase VO_2max_ more than MICT in healthy adults [42], and those with cardiometabolic diseases [14, 17, 43]. Increased VO_2_max and reduced resting heart rate can likely be attributed to a training-induced increase in stroke volume [44], which can be influenced by factors such as the reversal of left ventricular remodelling and increased ejection fraction (*via* increased end-diastolic volume and reduced end-systolic volume) [14]. Further, the improvement of systolic- and diastolic blood pressure and AIx in the boxing group indicates a reduction in total peripheral resistance (i.e. reversal of atherosclerosis) which is likely accompanied by improved endothelial function [45]. The underlying mechanism for these adaptations is the vascular shear stress induced by higher intensity exercise [46]. Numerous studies have shown that central and peripheral adaptations to exercise in sedentary and chronically diseased cohorts are superior with HIIT (on cycle or treadmill) as compared to MICT [13, 16, 18]. The changes experienced by the boxing group in the present study may be clinically significant, and therefore require further investigation.

### HRQoL outcomes

The boxing group increased Vitality (+54.8%; p = 0.024), Physical Functioning (+4.7%; p = 0.042) and General Health (+25.2; p = 0.07) domains of HRQoL with a large effect (Cohen’s *d* = 0.74-1.45) over time. By contrast, the walking group experienced a significant reduction in Vitality (-19.1%; p = 0.043) and experienced no change in the other two domains. A significant group x time effect favouring the boxing group was noted for Vitality (p = 0.02). Few studies have investigated the effect of HIIT on HRQoL. Significant improvements in SF-36 Physical Functioning and General Health domains of HRQoL have been noted in a previous study prescribing 12 weeks of HIIT performed on a treadmill in 88 men and women with essential hypertension [18]. Further, a study by Wisloff et al. [13], showed a statistically significant increase in the global domain of HRQoL in heart failure patients prescribed 12 weeks of HIIT versus MICT performed on treadmill. Our findings suggest that boxing training is effective for increasing several domains of HRQoL. There is a need to further explore the potential physiological mechanisms that contribute to the improvement of HRQoL and related psychological outcomes (e.g. emotion, motivation, life satisfaction, etc.) in response to HIIT in individuals who are sedentary and obese. Further, studies have shown that low HRQoL is associated with obesogenic dietary behaviours and work-productivity losses [47]; therefore, HIIT may be able to positively adapt these outcomes as well. Accordingly, robust RCT are required to further investigate the effects of boxing training on HRQoL and the clinical significance of these data.

## Conclusion

Boxing training (HIIT) in adults with abdominal obesity is feasible and may elicit a better therapeutic effect on obesity, cardiovascular- and HRQoL outcomes than an equivalent dose of brisk walking (MICT). Robustly designed randomized controlled trials are required to confirm these findings and inform clinical guidelines and practice for obesity treatment.
